# Effectiveness of Response Inhibition Training and Its Long-Term Effects in Healthy Adults: A Systematic Review and Meta-Analysis

**DOI:** 10.3389/fnins.2022.813975

**Published:** 2022-05-30

**Authors:** Wanyue Li, Yaru Shang, Weisheng Zhuang, Wangxiang Mai, Wenwen Cheng, Zhuoming Chen

**Affiliations:** ^1^Department of Rehabilitation, The First Affiliated Hospital of Jinan University, Guangzhou, China; ^2^Department of Rehabilitation, Henan Provincial People’s Hospital, People’s Hospital of Zhengzhou University, Zhengzhou, China; ^3^Department of Neurology, Maoming People’s Hospital, Maoming, China

**Keywords:** response inhibition training, meta-analysis, randomized controlled trial, long-term effects, healthy adults

## Abstract

**Objective:**

This study aims to evaluate the effectiveness and long-term effects of response inhibition training as a therapeutic approach in healthy adults.

**Methods:**

The PubMed, Embase, Web of Science, China National Knowledge Infrastructure (CNKI), Wanfang, and China Science and Technology Journal Database (VIP) were searched for studies. Data on the improvement of Cognitive function and its long-term effect were extracted by two authors independently. The pooled data were meta-analyzed using a random-effects model, and the quality of each eligible study was assessed by The Cochrane Collaboration’s tool.

**Results:**

Nine articles were included. 1 of the articles included 2 trials, so 10 eligible trials (response inhibition training group vs. control group) were identified. A total of 490 patients were included. Response inhibition training has beneficial effects on improving cognitive function in healthy adults compared to control treatment (SMD, −0.93; 95% CI, −1.56 to −0.30; *Z* = 2.88, *P* = 0.004), the subgroup analysis results showed that either GNG training alone (SMD, −2.27; 95% CI, −3.33 to −1.21; *Z* = 4.18, *P* < 0.0001) or the combination of both SST and GNG significantly improved cognitive function in healthy adults (SMD, −0.94; 95% CI, −1.33 to −0.56; *Z* = 4.80, *P* < 0.0001), whereas SST training alone did not have such an effect (SMD, −0.15; 95% CI, −0.76 to 0.47; *Z* = 0.47, *P* = 0.64). But its long-term effects are not significant (SMD, −0.29; 95% CI, −0.68 to 0.10; *Z* = 1.45, *P* = 0.15). The subgroup analysis results showed that neither GNG training alone (SMD, −0.25; 95% CI, −0.75 to 0.24; *Z* = 0.99, *P* = 0.32) nor SST training alone (SMD, 0.03; 95% CI, −0.42 to 0.48; *Z* = 0.14, *P* = 0.89) could improve the cognitive function of healthy adults in the long term. In contrast, the combination of both training (SMD, −0.95; 95% CI, −1.46 to −0.45; *Z* = 3.68, *P* = 0.0002) can have long-term effects on the improvement of cognitive function in healthy adults.

**Conclusion:**

The findings of our study indicate that response inhibition training can improve the cognitive function of healthy adults and that more RCTs need to be conducted to validate their usefulness in clinical cases.

## Introduction

Response inhibition can be conceptualized as the ability to stop, change or delay a behavioral response (Logan et al., 1984; Miyake et al., 2000; Bickel et al., 2012; Baumeister, 2014). It is one of the core components of executive function, and the ability to actively suppress, interrupt or delay behavior (Kooijmans et al., 2000; Groman et al., 2009; Muraven, 2010; Brydges et al., 2012). Individuals inhibit dominant responses formed through inhibitory control to flexibly adapt to changing environments while excluding or reducing the impact of irrelevant information on current information processing (Aron et al., 2004, 2007; Hofmann et al., 2009; Houben and Wiers, 2010; Nederkoorn et al., 2010; Murphy and Garavan, 2011; Verbruggen et al., 2014; Turton et al., 2017).

The research paradigm of response inhibition mainly includes Go/No-go tasks (GNG) and stop-signal tasks (SST). The consistent pairing of the no-go response with the target stimulus facilitated retrieval of the no-go target stimulus association and resulted in improved response inhibition to the target stimulus (Donders, 1969; Maguire and France, 2019). For example, Houben and Jansen (2011) used a GNG task with an alcohol-related stimulant in an attempt to reduce alcohol consumption. Participants in the training condition reported reduced alcohol consumption after training compared to the control condition, suggesting that an association was formed between the alcohol stimulus and the no-go response, which transferred to a reduction in alcohol consumption (Houben and Jansen, 2011). In the SST training paradigm, participants were asked to classify target and neutral stimuli as quickly as possible, however, in a subset of trials, the stop signal would appear after the target stimulus, and participants were asked to suppress their responses (Lawrence et al., 2015). In this way, an association between the target stimulus and the stop response was established. In the control condition, the stop signal was not always paired with a particular type of stimulus or was not presented at all. Lawrence et al. (2015) demonstrated that participants who received SST training in which stop-signals were paired with unhealthy foods consumed significantly less high-calorie food immediately after training, compared to those in the control condition. This suggests that establishing an association between unhealthy food and a stop response results in a reduction in the consumption of unhealthy foods.

In a meta-analysis by Jones et al. (2016), participants learn to associate appetitive cues with inhibition of behavior. The meta-analysis demonstrated that a single session of inhibitory control training (ICT) leads to a robust reduction in food and alcohol consumption in the laboratory. The effect of ICT on behavior was dependent on the task used: the effect was robust when modified GNG tasks were used and was marginally significant when Stop Signal tasks were used. In another study by Allom et al. (2015), results suggest that go/no-go inhibitory control training paradigms can influence health behavior, but perhaps only in the short term. Neither of the interventions included in the meta-analysis of these two studies was response inhibition training, Allom et al. (2015) used interventions for modified response inhibition training, Jones et al. (2016), the study population was also not all normal, including heavy drinkers, overly obese people, and addicted smokers. In contrast, there is a lack of research on the effectiveness and long-term effects of response inhibition training in improving cognitive function in healthy adults. However, most of the subjects used in the above studies had significant cognitive deficits, and response inhibition training is likely to significantly improve cognitive impairment in such individuals (Spierer et al., 2013; van Koningsbruggen et al., 2013; Jones et al., 2016), while it is controversial whether response inhibition training has an ameliorating effect on cognitive impairment in healthy adults. There is evidence of no real training and transfer effects after inhibitory, controlled training in young healthy adults (Enge et al., 2014). It has also been shown that response inhibition training can improve cognitive function in healthy adults (Ji et al., 2016). Cognitive interventions in children during their intellectual development and older populations can help the development of intellectual and other abilities and have, therefore, necessitated this review and meta-analysis to assess the effectiveness and long-term effects of response inhibition training as a treatment in healthy adults.

## Methods

Ethical approval and patient consent were not required because this was a systematic review and meta-analysis of previously published studies (Higgins and Thompson, 2002). This meta-analysis and systematic review were conducted based on the Preferred Reporting Items for Systematic Reviews and Meta-Analyses (PRISMA; Moher et al., 2009) guidelines and a previously published protocol (PROSPERO: CRD42021277898).

### Search Strategy

Any articles published before June 2021 in PubMed, Embase, Web of Science, China National Knowledge Infrastructure (CNKI), Wanfang, and China Science and Technology Journal Database (VIP) were searched using the following keywords: “Inhibition OR Suppression OR Interference Inhibition OR Go/No-go OR stop-signal tasks OR SST AND Training.” We manually searched for further literature by tracing the references included in the articles. We searched for papers published in English and Chinese. The inclusion criteria are presented as follows: (1) study design is RCT, (2) interventional studies that focus on response inhibition training, (3) including healthy participants older than 18 years, (4) reporting pre-training and follow-up assessment on at least one outcome measure.

### Data Extraction and Outcome Measures

We extracted the following information: author, year of publication, sample size, evaluation methodology, intervention methods, intervention frequency, Intervention intensity, and intervention time. Data were extracted independently by 2 investigators, and discrepancies were resolved by consensus. We applied a Java program called Plot Digitizer^1^ to convert plotted values into numerical form if adequate information was not provided by a study. We also contacted the corresponding author to obtain the data when necessary. The outcome indicators of this study were the degree of improvement in cognitive ability and the long-term effect outcome, the former including the scores of the included literature assessment tasks such as Go RT (ms), Go/no-go (RT), Stroop interference score (ms), go/no-go (IES), Working memory, Go RTs (ms), and the latter including the scores of the different period time assessment tasks.

### Quality Assessment

All eligible studies were assessed by two other authors independently. A third reviewer arbitrated in cases of disagreement. The Cochrane Risk of Bias Tool was used to assess the risk of bias of included RCTs (Higgins et al., 2008; Higgins and Green, 2008). We assessed seven types of bias accordingly, namely selection biases (random sequence generation and allocation concealment), blinded (performance bias and detection bias), attrition bias (incomplete outcome data), reporting bias (selective outcome reporting), and other biases. We categorized the risk of bias for each item as low, unclear, or high.

### Statistical Analysis

We estimated the standardized mean difference (SMD) and 95% confidence interval (CI) for the continuous outcome Cognitive function scores [Go RTs in ms, Go/no-go (RT), Stroop interference score (ms), go/no-go (IES), Working memory] using a random-effects model. Heterogeneity was reported using the *I*^2^ statistic, with an *I*^2^> 50% indicating significant heterogeneity. When significant heterogeneity emerged, we searched for potential sources of heterogeneity by sequentially omitting a study. Meta-analyses were performed or subgroup analyses were performed according to the different interventions (GNG/SST/GNG and SST). All statistical analyses were performed using Review Manager version 5.3 (Higgins and Thompson, 2002) (The Cochrane Collaboration, Software Update, Oxford, United Kingdom).

## Results

### Literature Search, Study Characteristics, and Quality Assessment

**FIGURE 1:**
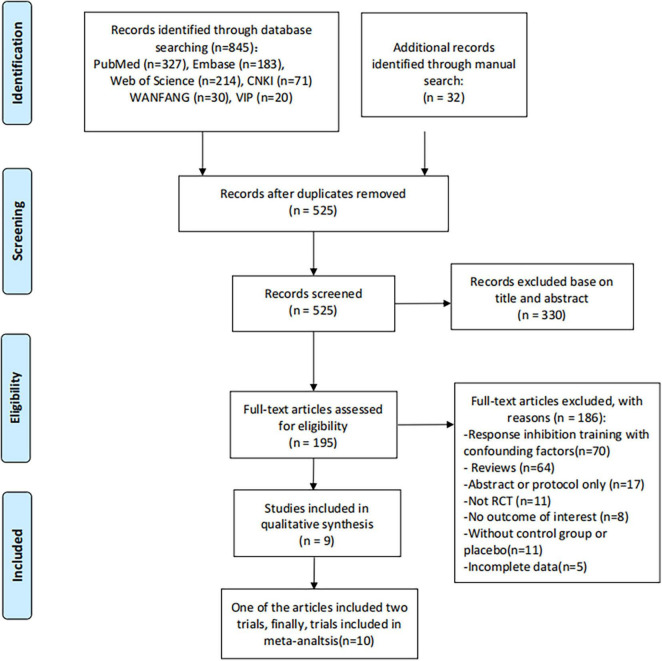
Flow diagram of study searching and selection process.

Figure 1 shows the detailed flowchart of the search and selection results. A total of 877 potentially relevant articles were identified initially. Finally, In the end, nine articles (Berkman et al., 2014; Enge et al., 2014; Allom and Mullan, 2015; Chen, 2016; Ji et al., 2016; Zhao et al., 2018; Schroder et al., 2020; Wang et al., 2020; Xu et al., 2021) were included, two articles in Chinese, seven articles in English. 1 of the articles included 2 trials, so there were 10 trials in the final meta-analysis. The baseline characteristics of the 10 eligible RCTs in the meta-analysis are summarized in Table 1. The total sample size included was 490. The 10 trials were all RCT studies (response inhibition training versus control group), two of which were blank controls, and the remaining eight studies were placebo training controls (Figure 1).

**TABLE 1:** Summary of the demographic and clinical characteristics of the included RCT studies (general response inhibition training vs. control group).

Author	n (Exp/Ctr)	Intervention		Assessment tasks	Intervention time
		
		Exp	Ctr		
Allom and Mullan, 2015	21/25	SST, each block consisted 64 trials. 1 time/day	Placebo training, the same task as the experiment group, no stop-signals 1 time/day	Stroop interference score (ms)	10 days
Allom and Mullan, 2015	23/23	SST, each block consisted 64 trials. 1 time/day	Placebo training, the same task as the experiment group, no stop-signals 1 time/day	Stroop interference score (ms)	10 days
Berkman et al., 2014	30/30	SST, 128 trials per time, 10 sessions that occurred approximately every other day for 3 weeks	Psychological training courses at the same time as the training group	SST, Go RT	23 days
Chen, 2016	23/23	GNG, 20 min/day, 20 days in total	Sand painting assignment, 20 min/day, 20 days in total	Go/No-go (IES)	20 days
Enge et al., 2014	38/38	15 min SST and 15 min GNG, 3 times/week, 9 times in total	Did not receive training or any other task	Go/no-go (RT)	3 weeks
Ji et al., 2016	18/16	SST, three computerized inhibition tasks and one group based inhibition game, the exact time is unknown. 3 times/week, 12 times in total	Mental health lectures, lectures on mental health once per week for four consecutive weeks. 45–60 min per session	Working memory	4 weeks
Schroder et al., 2020	23/24	GNG, 20 min/day, 4 days in total	Episodic memory training, 20 min/day, 4 days in total	Go RTs (ms)	3 days
Wang et al., 2020	27/22	SST without instant feedback, 800 trials per time, 3/week, 3 weeks in total	Did not receive training or any other task	Go/No-go (RT)	3 weeks
Xu et al., 2021	20/20	GNG and SST, 400 GNG and 200 SST per time, 3/week, 3 weeks in total	Read popular science articles, 30 min per time, 3/week, 3 weeks in total	Stroop interference score (ms), erp	3 weeks
Zhao et al., 2018	23/23	GNG, 600 trials per time, 20 min/day, 20 days in total	Sand painting assignment	Go/no-go (IES)	20 days

*IES, inverse efficiency score; GNG, Go/no-go; SST, stop-signal tasks; Exp, experiment group; Ctr, control group; erp, event-related potential.*

### Risk of Bias Assessment

**FIGURE 2:**
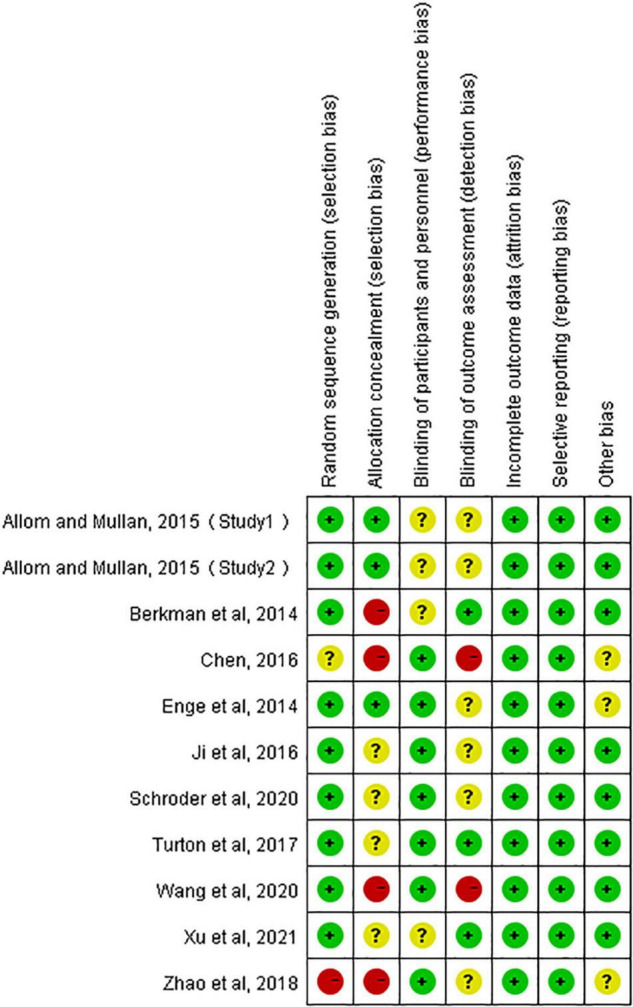
Risk of bias summary of RCTs. +, low risk; −, high risk; ?, unclear risk.

Chen (2016) and Wang et al. (2020) did not account for the allocation concealment (selection bias) and blinding of outcome assessment (detection bias). Berkman et al. (2014) did not account for the allocation concealment (selection bias). Zhao et al. (2018) did not account for random sequence generation (selection bias) and allocation concealment (selection bias). The remaining six studies were high-quality studies (Figure 2).

### Outcomes: Cognitive Function

**FIGURE 3:**
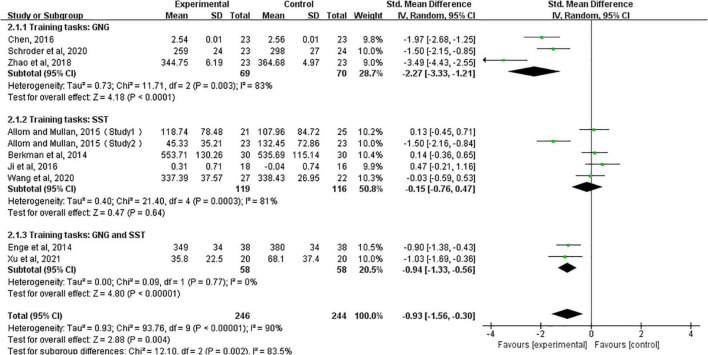
Immediate effects after training, based on subgroup forest plots for different training tasks (GNG, Go/no-go tasks; SST, stop-signal tasks; GNG and SST, Go/no-go tasks and stop-signal tasks).

These outcome data were analyzed with the random-effects model, Compared to controls, response inhibition training significantly improved cognitive function in healthy people (SMD, −0.93; 95% CI, −1.56 to −0.30; *Z* = 2.88, *P* = 0.004) with high heterogeneity (*I*^2^ = 90%), so we conducted subgroup analyses based on different response inhibition training (GNG, Go/no-go tasks; SST, stop-signal tasks; GNG and SST, Go/no-go tasks and stop-signal tasks). The results showed that either GNG training alone (SMD, −2.27; 95% CI, −3.33 to −1.21; *Z* = 4.18, *P* < 0.0001) or the combination of both SST and GNG significantly improved cognitive function in healthy adults (SMD, −0.94; 95% CI, −1.33 to −0.56; *Z* = 4.80, *P* < 0.0001), whereas SST training alone did not have such an effect (SMD, −0.15; 95% CI, −0.76 to 0.47; *Z* = 0.47, *P* = 0.64) (Figure 3).

### Sensitivity Analysis

**FIGURE 4:**
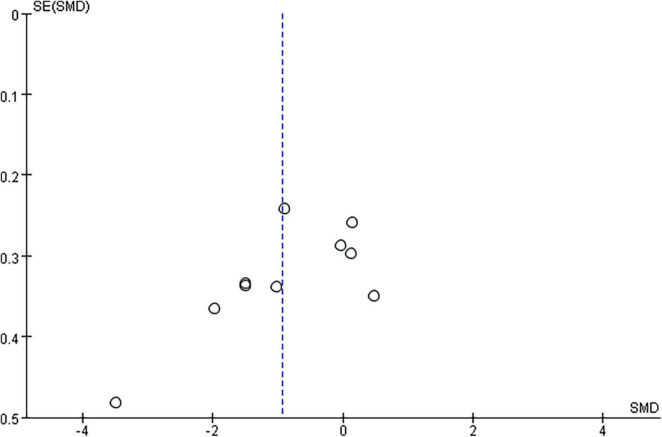
Funnel plots for the effect of Cognitive function. Blue color dotted line, the combined standardized mean difference (SMD) value.

Since the forest plot showed a large heterogeneity of studies, we used the literature-by-exclusion method for sensitivity analysis and found no literature with significant sources of heterogeneity. The funnel plot showed that the study by Zhao et al. (2018) was far from the mid-line, and the sensitivity analysis did not significantly reduce after removing this article. Therefore, we did not exclude this article (Figure 4).

### Long-Term Effects

**FIGURE 5:**
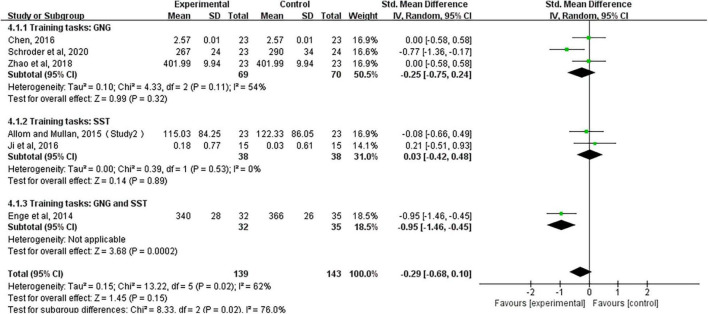
Long-term follow-up effects after training, based on subgroup analysis forest plots for different training tasks (e.g., GNG, Go/no-go tasks; SST, stop-signal tasks; GNG and SST, Go/no-go tasks and stop-signal tasks).

A meta-analysis of long-term follow-up results showed that response inhibition training did not improve cognitive function in healthy adults in the long term (SMD, −0.29; 95% CI, −0.68 to 0.10; *Z* = 1.45, *P* = 0.15), but meta-analysis results were highly heterogeneous (*I*^2^ = 62%), so we conducted subgroup analyses based on different response inhibition training (GNG, SST, GNG, and SST). The results showed that neither GNG training alone (SMD, −0.25; 95% CI, −0.75 to 0.24; *Z* = 0.99, *P* = 0.32) nor SST training alone (SMD, 0.03; 95% CI, −0.42 to 0.48; *Z* = 0.14, *P* = 0.89) could improve the cognitive function of healthy adults in the long term. In contrast, the combination of both trainings (SMD, −0.95; 95% CI, −1.46 to −0.45; *Z* = 3.68, *P* = 0.0002) can have long-term effects on the improvement of cognitive function in healthy adults (Figure 5).

### Publication Bias

In this analysis, there was no publication bias on the Egger test for cognitive function (*P* = 0.052) and long-term effects (*P* = 0.148).

## Discussion

The main results of this systematic review and meta-analysis are as follows: (1) Compared with the control treatments, response inhibition training benefits improving cognitive function in healthy adults but its long-term effects are not significant. (2) Immediate results after treatment showed that either GNG training alone or the combination of both SST and GNG significantly improved cognitive function in healthy adults, whereas SST training alone did not have such an effect. (3) Long-term follow-up efficacy after treatment showed that neither GNG training alone nor SST training alone could improve the cognitive function of healthy adults in the long term. In contrast, the combination of these two training can have a long-term impact on the improvement of healthy adult cognitive functions.

The effectiveness of response inhibition training has been widely debated for a long time. The results of a meta-analysis by Jones et al. (2016) showed that food-specific inhibition training can improve the eating behavior of obese, binge eaters in the short term, and another study by Allom et al. (2015), showed that the Go/No-Go inhibitory control training paradigm can influence the health behavior of excessive smokers and drinkers, but perhaps only in the short term. Neither of the interventions included in the meta-analysis of these two studies was response inhibition training, the intervention used by Allom et al. (2015) was modified response inhibition training, and Jones et al. (2016), nor was the study population entirely normal, including heavy drinkers, overly obese individuals, and addicted smokers. In contrast, there is a lack of research on the effectiveness and long-term effects of response inhibition training in general in improving cognitive function in healthy adults. There is evidence that there is no real training and transfer effect after response inhibition training in healthy adults (Enge et al., 2014). There is also evidence that response inhibition training, in general, improves cognitive function in healthy adults (Ji et al., 2016). Therefore, to resolve this contradiction our search included only unmodified response inhibition training and the inclusion population was selected only from healthy adults to exclude the interference of conditions such as modified response inhibition training, alcohol abuse, excessive smoking, excessive obesity, and to assess the effectiveness and long-term effects of response inhibition training alone on cognitive function improvement in healthy adults.

Overall, training using GNG seems to produce larger effects than training using SST, suggesting that the two tasks may have different mechanisms that lead to different effects on behavior. Indeed, previous studies have confirmed this hypothesis, and the GNG and SST involve different functional bases and neural mechanisms. The Go/No go task and the stop-signal task training involve a functional basis of auto-activated inhibition and top-down control inhibition, respectively, and both have the same neural pathway of the sensory cortex-sub frontal gyrus-basal ganglia-thalamus-primary motor cortex, but the speed of action is different due to the different functional basis involved. Go/No go task training has a stable stimulus-response (S-R) mapping that establishes automatic activation pathways and faster inhibition; stop-signal van task training has a continuously changing S-R mapping that requires top-down cognitive control involvement and slower inhibition (Spierer et al., 2013). Therefore, preliminary evidence found that GNG training influences behavior through bottom-up response inhibition (Verbruggen and Logan, 2008). On the other hand, SST training may contribute to improved reactive control. Allom and Mullan (2015) sought to identify the underlying mechanisms of SST training, arguing that training improves Stroop performance, which is arguably a measure needed to control for response inhibition (Friedman and Miyake, 2004). However, although Stroop scores improved in those who received SST training, this did not translate into improvements in cognitive behavior. It may be the case that GNG and SST affect behavior through automatic and controlled response inhibition, respectively, but training automatic response inhibition may be more effective for behavior change.

In the follow-up efficacy analysis, neither SST nor GNG training alone was found to improve cognitive function in healthy adults, whereas the combination of the two had significant long-term effects. This gives us a new insight that SST combined with GNG training may have a more long-term, robust effect on improving the cognitive function in healthy adults. However, the mechanism needs to be confirmed by further studies. Response inhibition, the ability to regulate and inhibit human behavior so that it immediately responds to the most motivating stimuli in the environment, is a very important human ability. Individuals with strong response inhibition are better able to resist undesirable behaviors, including dietary fat intake and sleep hygiene, as well as addictive behaviors including alcohol consumption and excessive smoking (Friedman and Miyake, 2004; Hofmann et al., 2009; Houben and Wiers, 2010; Nederkoorn et al., 2010; Murphy and Garavan, 2011). Therefore, combined with the results of our study, we believe that SST combined with GNG training has social benefits and economic benefits and is worthy of clinical promotion and application. Moreover, inhibition training is one of the intervention methods to improve cognitive function, it works by reinforcing a certain cognitive process. Therefore, the duration of training certainly affects the long-term effects of inhibition training. Whereas due to the fatigue effect or ceiling effect, we claimed that the effect caused by training time is not increased indefinitely at any time but remains stable after reaching a certain level, and more research is needed to confirm this issue.

Since the forest plot showed a large heterogeneity of studies, we used the literature-by-exclusion method for sensitivity analysis and found no literature with significant sources of heterogeneity. We performed a subgroup analysis based on different intervention methods, and the heterogeneity could be explained by the small number of included literature, the small sample size, national, and geographical differences. Several limitations exist in this meta-analysis. First, the sample sizes of the 10 studies we included were relatively small (*n* < 100), and more RCTs with large patient samples should be conducted to explore this issue. Although this study collected and analyzed literature through various academic databases, it only evaluated publications written in English and Chinese. Finally, some unpublished and missing data may lead to some bias for the pooled effect.

In conclusion, response inhibition training is a benefit for improving cognitive function in healthy adults but its long-term effects are not significant, whereas the combination of both trainings can have long-term effects on the improvement of cognitive function.

## Author Contributions

ZC and WC took responsibility for the integrity of data and the accuracy of their analysis. WL and YS contributed to the conception and design of the study and writing of the manuscript. WZ prepared the figures and tables. WL, WM, YS, ZC, WZ, and WC contributed substantially to the literature search, data extraction and analysis, data interpretation, and quality assessment. All authors contributed to the article and approved the submitted version.

## Conflict of Interest

The authors declare that the research was conducted in the absence of any commercial or financial relationships that could be construed as a potential conflict of interest.

## Publisher’s Note

All claims expressed in this article are solely those of the authors and do not necessarily represent those of their affiliated organizations, or those of the publisher, the editors and the reviewers. Any product that may be evaluated in this article, or claim that may be made by its manufacturer, is not guaranteed or endorsed by the publisher.

## Footnotes

1
http://plotdigitizer.sourceforge.net
